# Synthetic Artificial Long Non-coding RNA Shows Higher Efficiency in Specific Malignant Phenotype Inhibition Compared to the CRISPR/Cas Systems

**DOI:** 10.3389/fmolb.2020.617600

**Published:** 2020-12-09

**Authors:** Lin Yao, Quan Zhang, Aolin Li, Binglei Ma, Zhenan Zhang, Jun Liu, Lei Liang, Shiyu Zhu, Ying Gan, Qian Zhang

**Affiliations:** ^1^Department of Urology, Peking University First Hospital, Beijing, China; ^2^Institute of Urology, Peking University, Beijing, China; ^3^National Research Center for Genitourinary Oncology, Beijing, China

**Keywords:** bladder cancer, synthetic biology, aptamer, miRNA, transcription factors

## Abstract

**Objective:** Both oncogenic transcription factors (TFs) and microRNAs (miRNAs) play an important regulator in human cancer by transcriptional and post-transcriptional regulation, respectively. These phenomena raise questions about the ability of artificial device to regulate miRNAs and TFs simultaneously. In this study, we aimed to construct an artificial long non-coding RNA, “alncRNA,” which imitated CRISPR/Cas systems and to illuminate its therapeutic effects in bladder cancer cell lines. At the same time, we also compared the efficiency of alncRNA and CRISPR/Cas systems in regulating gene expression.

**Study Design and Methods:** Based on engineering principles of synthetic biology, we combined tandem arrayed cDNA sequences of aptamer for TFs with tandem arrayed cDNA copies of binding sites for the miRNAs to construct alncRNA. In order to prove the utility of this platform, we chose β -catenin, NF-κB, miR-940, and miR-495 as the functional targets and used the bladder cancer cell lines 5637 and T24 as the test models. Real-time Quantitative PCR (qPCR), dual-luciferase assay and relative phenotypic experiments were applied to severally test the expression of relative gene and therapeutic effects of our devices.

**Result:** Dual-luciferase assay indicated alncRNA could inhibit transcriptional activity of TFs. What’s more, the result of qPCR showed that expression levels of the relative TFs target genes and miRNAs were reduced by corresponding alncRNA and the inhibitory effect was better than CRIPSR dCas9-KRAB. By functional experiments, decreased cell proliferation, increased apoptosis, and motility inhibition were observed in alncRNA-infected bladder cells.

**Conclusion:** In summary, our synthetic devices indeed function as anti-tumor regulator, which synchronously accomplish transcriptional and post-transcriptional regulation in bladder cancer cell and show higher efficiency in specific malignant phenotype inhibition compared to the CRISPR/Cas systems. Most importantly, Anti-cancer effects were induced by the synthetic alncRNA in the bladder cancer lines. Our devices, therefore, provides a novel strategy for cancer therapy and could be a useful “weapon” for cancer cell.

## Introduction

Bladder cancer (BCa) is a malignant tumor that occurs on the mucous membrane of the bladder. It is the most common malignant tumor in the urinary system and one of the ten most common tumors in the whole body (Kluth et al., 2015; Kamat et al., 2016; Siegel et al., 2017). At present, the treatment for BCa is mainly of surgical treatment, but the post-operative recurrence and metastasis of tumor are still important factors that threaten the life of patients (Racioppi et al., 2012). In addition, chemotherapy or radiotherapy can reduce some of the symptoms of BCa patients, they all have certain curative effect on BCa, but the adverse reactions are large, and these treatment methods cannot actually prolong the survival of patients (Marta et al., 2012; Sofra et al., 2013). There are several novel anti-tumor drugs have been developed and clinically testing their effectiveness, however, these drugs can only be used in the adjuvant treatment of BCa (Wong et al., 2017). It is therefore necessary to develop new treatment methods for BCa. The molecular targeted therapy of tumor is a novel and effective way to improve the therapeutic effect of BCa.

Research on molecular biology confirms that the occurrence and development of malignant tumor is a multi-factor and multi-step process, and the development of BCa is more so (Hanahan and Weinberg, 2011). miRNAs are non-coding RNAs of approximately 19–23 nucleotides lengths, and like other small RNAs, miRNAs have been considered as the byproducts of transcription in the past (Djebali et al., 2012). With the deepening of the research on miRNAs, miRNAs had been found to have an effect on the biological physiological and pathological changes (Rupaimoole and Slack, 2017). As a type of endogenous small single-stranded non-coding RNA, miRNAs regulate the translation and stability of mRNAs through complementary binding with the 3′UTR of mRNAs and play a role in post-transcriptional regulation (Bracken et al., 2016). Although the number of miRNAs is relatively small, more than 1/3 of the cell transcriptional groups are regulated by miRNAs. In addition, the dysregulation of miRNAs expression is an important mechanism to promote the occurrence and development of tumors, and so is the human BCa. Previous work has shown that miR-495 can target phosphatase and tensin homolog and promote the proliferation and invasion of BCa cells through its interaction with phosphatase and tensin homolog (Tan et al., 2017). miR-940 plays a role in promoting the progression of human BCa by activating the c-myc protein, and its upregulation has been proved to serve as an effective biomarker that predicts the poor prognosis for BCa (Wang et al., 2018). Thus, regulating the dysregulation of miRNAs and reprogramming the signaling pathway in BCa cells will effectively control the malignant phenotype of BCa cells and achieve the purpose of treating BCa.

Transcription factors (TFs) are presented in the nucleus and play a central role in the expression of gene within the cells (Haberle and Stark, 2018). In tumor cells, TFs affect the formation, evolution and metastasis of the tumor by regulating the expression of oncogenes, tumor suppressor genes and cell cycle related molecules. To date, some cancer-related TFs such as β-catenin (Lee et al., 2006) and NF-κB (Culler et al., 2010) have been proved to be significantly abnormal and highly expressed in BCa, which is highly correlated with the malignancy of the tumor. Therefore, it would be a further important attempt to inhibit tumorigenesis if these cancer-related TFs can be restrained from continuing to function in the tumor cells.

In this study, we constructed a working “artificial long non-coding RNA” (alncRNA) based on synthetic biology principles and tested its effectiveness in the BCa cell lines. The alncRNA we constructed is mainly composed of miRNAs binding sites and cancer-related TFs specifically binding sites. This alncRNA can simultaneously absorb and downregulate cancer-related miRNAs and protein with the BCa cells and achieved multiple targets and inhibiting tumor cells at the same time.

## Materials and Methods

### Cell Lines and Cell Culture

The human bladder cancer cell lines (T24, 5637, and SW780) and the human normal bladder epithelial cell line (SV-HUC1) are all bought from the American Type Culture Collection (ATCC, Manassas, VA, United States). In our study, we used two types of mediums, Dulbecco’s modified Eagle’s medium (DMEM, Gibco, Thermo Fisher Scientific, Waltham, MA, United States) and F12K medium (Gibco). The bladder cancer cell lines T24, 5637, and SW780 were grown in the DMEM, supplemented with 10% fetal bovine serum (Gibco). The SV-HUC1 cell line was grown in the F12K medium which was supplemented with 10% fetal bovine serum. All the cells were cultured at 37°C and 5% CO_2_.

### Construction of the Artificial Long Non-coding RNA and Cell Transfection

We linked the binding sites of miR-940 and miR-495 to construct the module 1 of the alncRNA. In addition, the aptamers for β-catenin and NF-κB were bulged by us to construct the module 2. All the nucleic acid sequences required in our study were gained by chemical synthesis.

### Dual-Luciferase Reporter Assay

The dual-luciferase reporter assay was performed to evaluate the transcriptional activity of β-catenin in our study. We constructed the dual-luciferase reporter vectors of β-catenin, respectively.

### RNA Extraction and Real-Time Quantitative PCR Assay

Total RNAs from cancer tissues or cells after transfection were extracted using the TRIzol reagent (Invitrogen, Grand Island, NY, United States) according to the manufacturer’s instructions. PrimeScript RT Reagent Kit with gDNA Eraser (Takara, Japan) was used to transform RNA to cDNA. The mRNA expression levels were measured by using SYBR^®^ Premix Ex Taq^TM^ (TaKaRa, Japan) according to the user’s manuals on the Roche lightcycler 480 Real-Time PCR System. GAPDH was utilized as the control to normalize the data. The primer sequences were listed in Table 1. The comparative ΔCt method was used to analyze the results by calculating the relative amount. All experiments were carried out at least three repetitions.

**TABLE 1:** Relative primers used in this research.

Name	Sequences
GAPDH-F	CGCTCTCTGCTCCTCCTGTTC
GAPDH-R	ATCCGTTGACTCCGACCTTCAC
β-catenin-F	CTGCTCTAGTAATAAGCCGGCT
(β-catenin-R	CAGGTGACCACATTTATATCAT
NF-(κB-F	AGAGGCGTGTATAAGGGGCT
NF-(κB-R	TTACTGTCATAGATGGCGTCTG
c-MYC-F	GGCTCCTGGCAAAAGGTCA
c-MYC-R	CTGCGTAGTTGTGCTGATGT
Cyclin D1-F	GCTGCGAAGTGGAAACCAT
Cyclin D1-R	CCTCCTTCTGCACACATTTGAA
BCL-XL-F	GAGCTGGTGGTTGACTTTCTC
BCL-XL-R	TCCATCTCCGATTCAGTCCCT
TRAF1-F	TCCTGTGGAAGATCACCAATGT
TRAF1-R	GCAGGCACAACTTGTAGCC
Vimentin-F	GACGCCATCAACACCGAGTT
Vimentin-R	CTTTGTCGTTGGTTAGCTGGT
Slug-F	CGAACTGGACACACATACAGTG
Slug-R	CTGAGGATCTCTGGTTGTGGT
E-cadherin-F	CGAGAGCTACACGTTCACGG
E-cadherin-R	GGGTGTCGAGGGAAAAATAGG

### Cell Proliferation Assay

In our study, we used CCK-8 assay (TransGen, Beijing, China) and Edu incorporation assay (Ribobio, Guangzhou, China) to determine cell proliferation. The operation steps of both experiments were mainly carried out according to the instructions of the manufacturer and adjusted through the operation steps of previous studies. Additionally, we have repeated these experiments for three times.

### Cell Apoptosis Assay

Flow cytometry assay and caspase-3 assay were used to determine the apoptosis level in our study. Aliquots of 1 × 106 cells were counted to measure the activity of caspase-3 by using caspase-3 ELISA assay kit (Biovision, Milpitas, CA, United States). The OD values were measured at 405 nm by using a microplate reader. As for flow cytometry assay, the flow cytometry assay kit (TransGen, Beijing, China) was used to evaluate the cell apoptosis level and the specific operation steps were performed according to the instructions of the manufacturer. Additionally, we have repeated these experiments for three times.

### Cell Motility Assay

The level of cell motility was measured by wound-healing assay. A sterile 200 μL pipette tip was used to create the wound field when cells grown reached approximately 85–90% confluence. A digital camera system was used to monitor the migration of cells. After the wound was created for 24 h, the migration distance (μm) of cells was measured by the software program (HMIAS-2000). We have repeated these experiments for three times. Additionally, the specific experimental procedure is mainly referred to the previous literature.

### Statistical Analysis

All the experiments were repeated five times. All the digital data were represented in the form of mean ± standard deviation (SD). SPSS 17.0 software (IBM Corp, Armonk, NY, United States) was used to perform the statistical analyses in our study. Statistical significance was tested by Chi square test, ANOVA and Student’s *t*-test. *P* < 0.05 was considered to be statistically significant.

## Results

### The Design and Construction of Artificial Long-Non-coding RNA for Suppressing BCa

The alncRNA we constructed that functions as a tumor suppressor is mainly composed of two modules, one of which is the module for downregulating the TFs that are related to the cancer, and the other is a module for down-regulation of miRNAs. The nucleic acid aptamers are small oligonucleotide sequences or short polypeptides screened and they can bind to the corresponding protein ligands with high affinity and strong specificity (Kwak et al., 2009). After the aptamers specifically binds to the protein ligands, the function of the protein will be inhibited (Culler et al., 2010; Liu et al., 2013). Inspired by the interaction between the aptamer and the protein ligand, we, respectively, connected two different aptamers to form the first module. In addition, some RNA elements can bind and regulate miRNAs based on the principle of base pairing. According to this principle, we designed the corresponding miRNA sponges and put them together in series to form the second modules.

**FIGURE 1:**
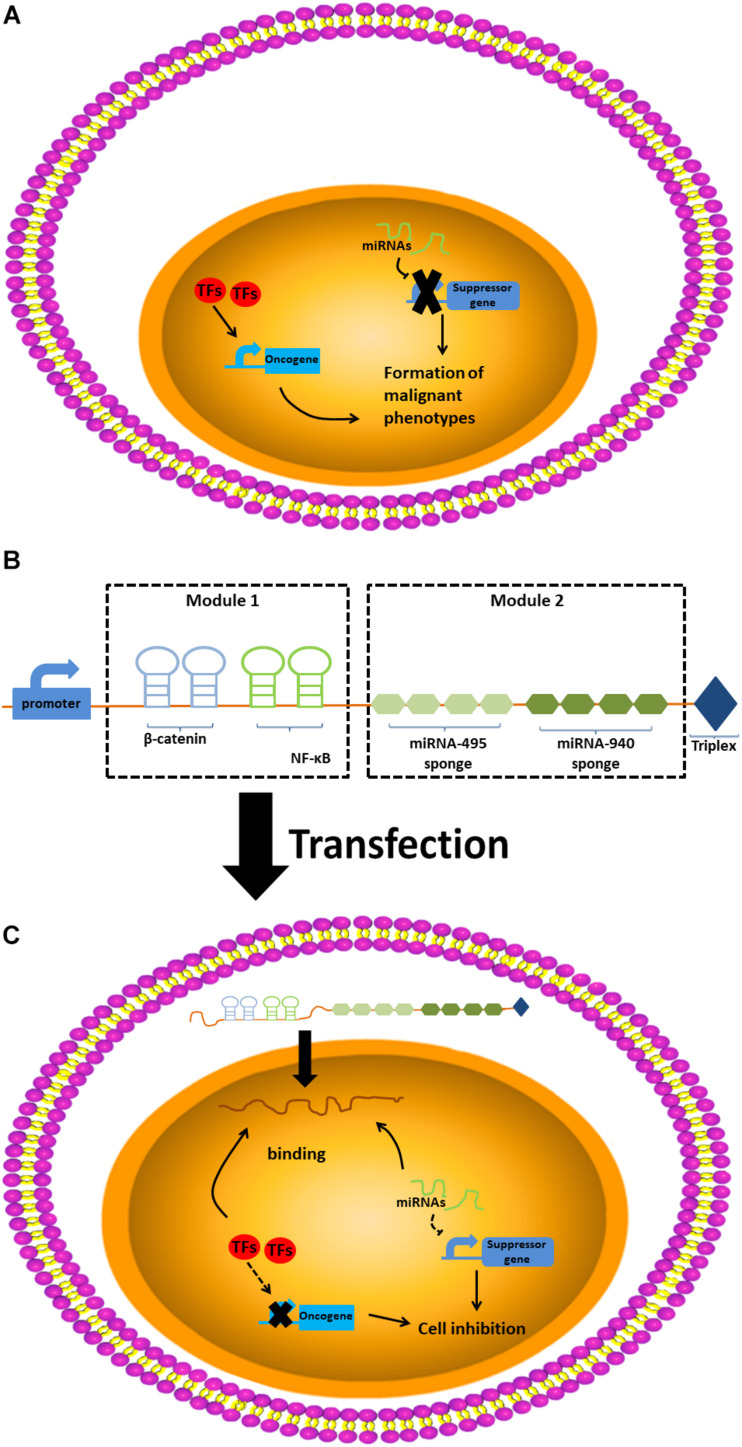
The design and construction of artificial long-non-coding RNA. **(A)** The design of artificial long-non-coding RNA. **(B)** The construction of artificial long-non-coding RNA. **(C)** The work pattern of artificial long-non-coding RNA.

In our study, we connected two aptamers for β-catenin (Tetsu and McCormick, 1999) and NF-κB in series to construct the first modules of the alncRNA we designed. In addition, after analyzing the sequences of miRNA-495 and miRNA-940, we designed the miRNA sponges sequence of these two miRNAs according to the principle of competing endogenous RNAs (ceRNAs). We connected five miRNA sponges for miRNA-495 and five miRNA sponges for miRNA-940 in series to construct the second modules of the alncRNA. Finally, we effectively combine the two modules to form the complete alncRNA. In order for the alncRNA to be stably present in the cells without degradation, we attached the tail of MALAT1 at the 3′ end of the alncRNA (Zhan et al., 2018; Figure 1).

### The alncRNA Inhibited the Oncogenic Factors β-Catenin in the BCa Cells

**FIGURE 2:**
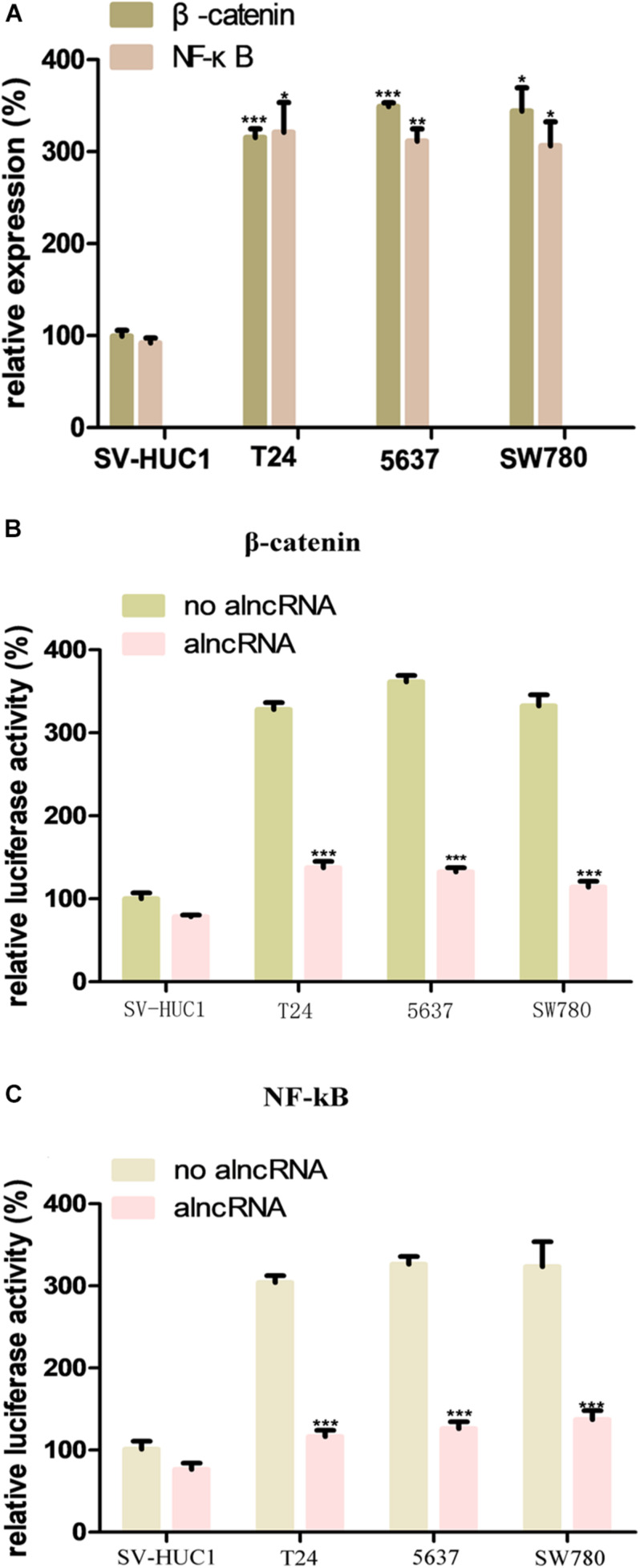
The oncogenic factors β-catenin and NF-κB is repressed by alncRNA in the BCa cells. **(A)** QRT-PCR analyses the expression levels of β-catenin and NF-κB in the normal bladder epithelial cell line (SV-HUC1) and BCa cell lines T24, 5637 and SW780. **(B)** The relative luciferase activity of β-catenin in the normal bladder epithelial cell line and BCa cell lines. **(C)** The relative luciferase activity of NF-κB in the normal bladder epithelial cell line and BCa cell lines. *p < 0.05, **p < 0.01, and ***p < 0.001.

β-catenin and NF-κB are important oncogenic transcription factors, and their abnormal upregulation has been found in the tumor cells, especially BCa cells (Liu et al., 2016). In our study, to investigate the expression levels of them in the BCa, we used qRT-PCR assay to measure their expression levels in the BCa cell lines T24, 5637, and SW780. Meanwhile, we used the normal bladder epithelial cell line (SV-HUC1) as the control group to verify the abnormal overexpression of theβ-catenin and NF-κB in the BCa cells. The results were as expected, the expression levels of β-catenin and NF-κB were significantly higher than those of the control group (Figure 2A).

We set the aptamers of β-catenin and NF-κB in the alncRNA and intended to use them to absorbed these oncogenic factors. In this way, the alncRNA we designed would serve as an anti-cancer factor which may effectively inhibit the function of β-catenin and NF-κB and inhibit the development of BCa. In order to validate that the alncRNA can indeed absorb β-catenin and NF-κB to as the anti-cancer factors, we used the dual-luciferase reporters assay to verify this effect. The responsive promoter sequences of β-catenin and NF-κB were specifically set into the dual-luciferase vectors, respectively, and we used these reporters to sense the transcriptional activity of β-catenin and NF-κB within the cells (Figures 2B,C).

Firstly, we transfected the two dual-luciferase reporters into the cell lines in our study to observe the feasibility of these two reporters. Results as expected, the transcriptional activity of β-catenin in the BCa cell lines (T24, 5637, and SW780) was decreased obviously after transfected with the alncRNA compared to negative control alncRNA (Figure 2B). Next, we explore whether the alncRNA we constructed can inhibit the transcriptional activity of NF-κB within the cells. Surprisingly, the alncRNA can obviously inhibit the transcriptional activity of NF-κB in the BCa cells, but when it comes to the control group, there was no significant change in the transcriptional activity ofβ-catenin (Figure 2C). It may be that in the normal cells, the expression level of β-catenin is low, and they mainly located in the cytoplasm rather than the nucleus, so the reporters is not sensitive to them in the normal cells. In conclusion, we have proved that the alncRNA we constructed can effectively down-regulate the transcriptional activity ofβ-catenin in the BCa cells.

### The alncRNA Inhibited the Oncogenic micoRNAs

**FIGURE 3:**
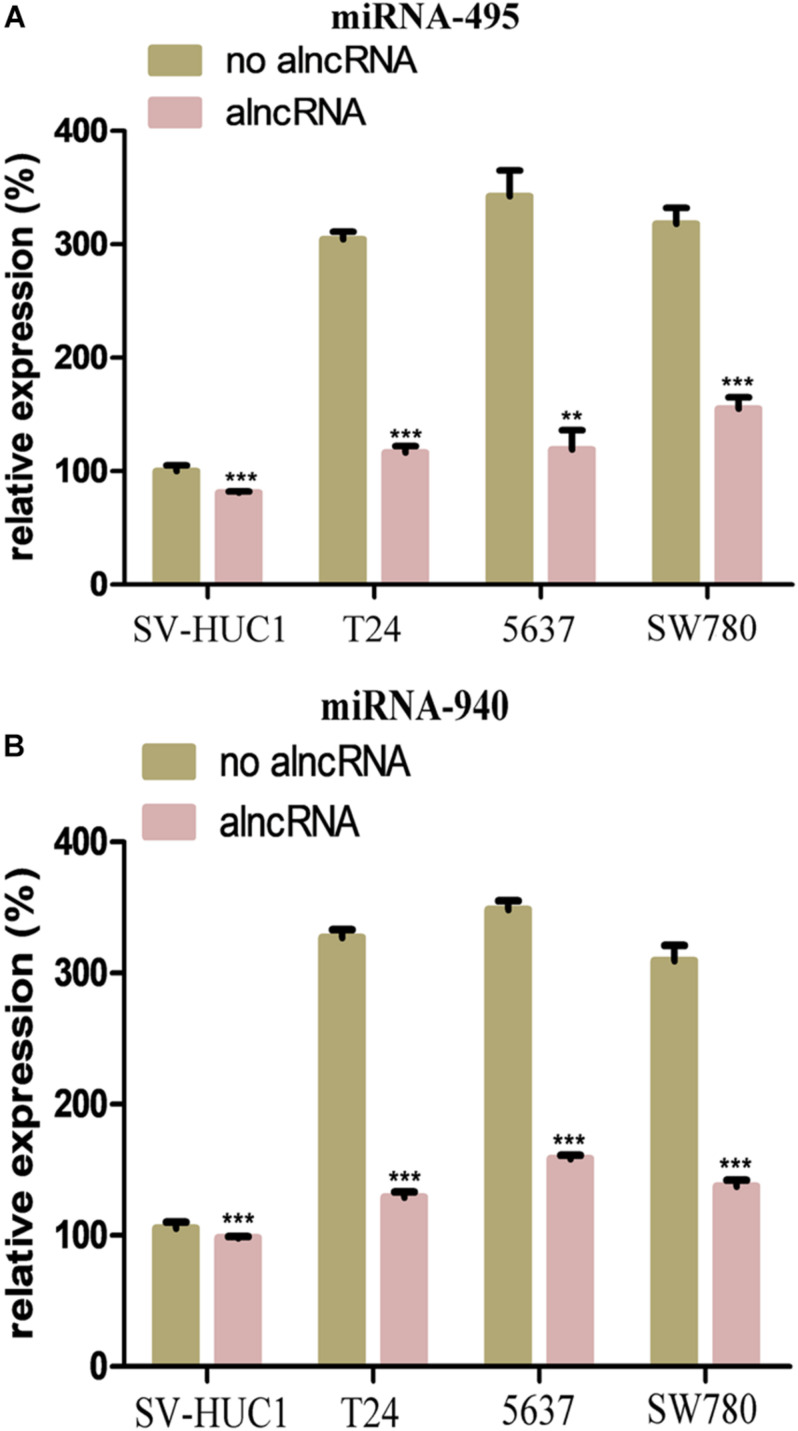
The alncRNA inhibited the oncogenic micoRNAs in the BCa cells. **(A)** QRT-PCR analyses the expression levels of miRNA-495 in the normal bladder epithelial cell line (SV-HUC1) and BCa cell lines T24, 5637 and SW780. **(B)** QRT-PCR analyses the expression levels of miRNA-940 in the normal bladder epithelial cell line (SV-HUC1) and BCa cell lines T24, 5637, and SW780. **p < 0.01 and ***p < 0.001.

In our study, we selected two oncogenic micoRNAs (miRNA-495 and miRNA-940) as the mainly targets of the module 2. To investigate the expression levels of miRNA-495 and miRNA-940 in the BCa cells, we also used the qRT-PCR assay to measure the relative expression of them in the T24, 5637, and SW780. Meanwhile, we used the SV-HUC1 as the control group. As expected, the expression levels of miRNA-495 and miRNA-940 in the BCa cell lines were higher than the normal baldder epithelial cell line (Figures 3A,B). After the transfection of the alncRNA, the expression level of the miRNA-495 and miRNA-940 in the cell lines were reduced to varying degrees (Figures 3A,B). Therefore, we believed that the alncRNA we constructed was indeed effective in downregulating the target micoRNAs (miRNA-495 and miRNA-940).

### The Inhibitory Effect of alncRNA Was Better Than CRISPR dCas9-KRAB

**FIGURE 4:**
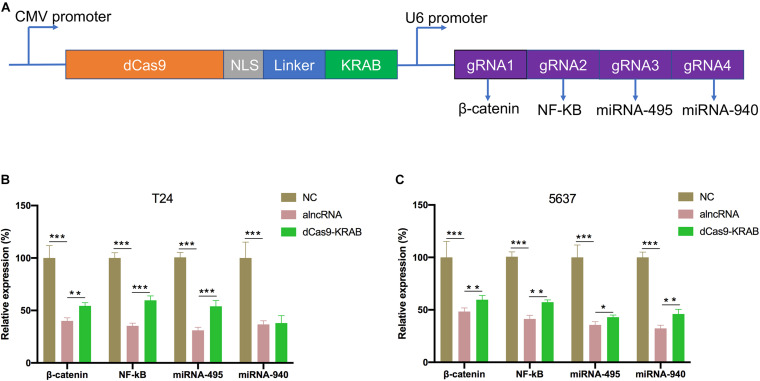
The inhibitory effect of alncRNA was better than CRISPR dCas9-KRAB. **(A)** The construction of dCas9-KRAB and the targets of sgRNA. **(B)** QRT-PCR analyses the expression levels of β-catenin, NF-κB, miRNA-495 and miRNA-940 in the normal bladder epithelial cell line (SV-HUC1) and BCa cell line (T24) after transfected with the alncRNA and dCas9-KRAB. **(C)** QRT-PCR analyses the expression levels of β-catenin, NF-κB, miRNA-495, and miRNA-940 in the normal bladder epithelial cell line (SV-HUC1) and BCa cell line (5637) after transfected with the alncRNA and dCas9-KRAB. *p < 0.05, **p < 0.01, and ***p < 0.001.

CRISPR/Cas9 genome engineering has revolutionized all aspects of biological research, including cancers, with epigenome engineering transforming gene regulation studies. Here, we would like to compare the inhibitory effect of alncRNA with CRISPR/Cas9. First, fusions of nuclease-inactive dCas9 to the Krüppel-associated box (KRAB) repressor (dCas9-KRAB) can silence target gene expression. So, we targeted dCas9-KRAB to the promoter of β-catenin, NF-κB, miRNA-495, and miRNA-940 (Figure 4A). Then we observed the expression levels of β-catenin, NF-κB, miRNA-495, and miRNA-940 were significantly decreased after transfected with the dCas9-KRAB. Unexpectedly, the results showed that the alncRNA showed a better inhibitory effect than dCas9-KRAB in T24 and 5637 BCa cell lines (Figures 4B,C).

### The alncRNA Effectively Inhibited the Downstream Oncogenic Signals

Next, we further investigated the action mechanism of the alncRNA to clarify its anti-cancer effect. We examined the relative expression of the downstream oncogenic signals of the target TFs and miRNAs in the BCa cell lines T24, 5637, and SW780. As we know from the previous qRT-PCR results, the alncRNA we constructed could significantly reduce the expression of the target oncogenic TFs and miRNAs. C-myc and cyclin D1 are the most important functional signaling molecules in the regulatory pathway ofβ-catenin and miRNA-940, while Bcl-XL and TRAF1 are the final signaling factors that are upregulated by miRNA-495 when they play a role in promoting the development of BCa. In theory, when we down-regulate these oncogenic TFs and miRNAs which are targeted by the alncRNA we constructed, these final effector factors will be inhibited. In this way, the alncRNA we constructed may effectively inhibit the malignant phenotype of the BCa cells and finally achieve the effect of treating BCa.

**FIGURE 5:**
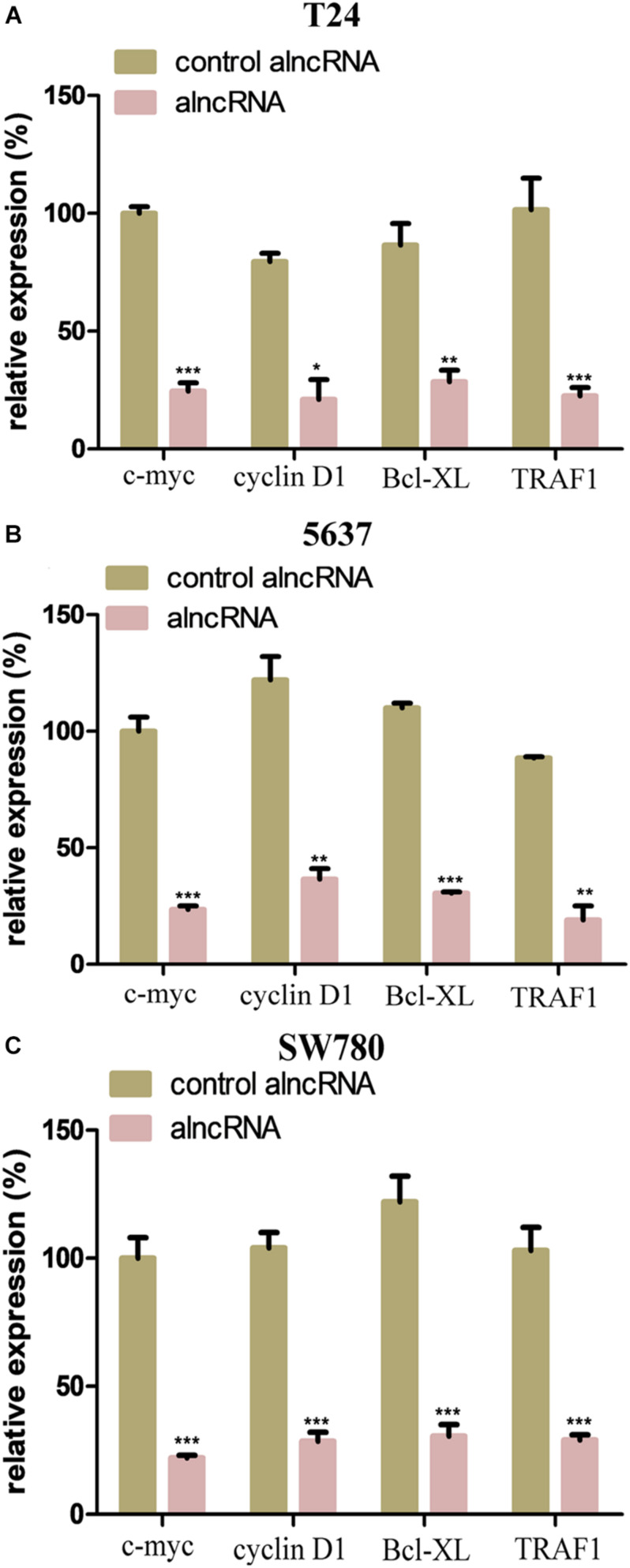
The alncRNA effectively inhibited the downstream oncogenic signals. **(A)** QRT-PCR analyses the expression levels of the downstream oncogenic signals in BCa cell line (T24). **(B)** QRT-PCR analyses the expression levels of the downstream oncogenic signals in BCa cell line (5637). **(C)** QRT-PCR analyses the expression levels of the downstream oncogenic signals in BCa cell line (SW780). *p < 0.05, **p < 0.01, and ***p < 0.001.

We mainly used qRT-PCR assay to determine whether the alncRNA could successfully down-regulate these important oncogenic signaling molecules (c-myc, cyclin D1, Bcl-XL, and TRAF1). As shown in the Figure 5, we found that the alncRNA can actually down-regulate the expression of c-myc, cyclin D1, Bcl-XL, and TRAF1in the BCa cell lines.

### The alncRNA Induced the Cell Apoptosis of BCa Cell Lines

**FIGURE 6:**
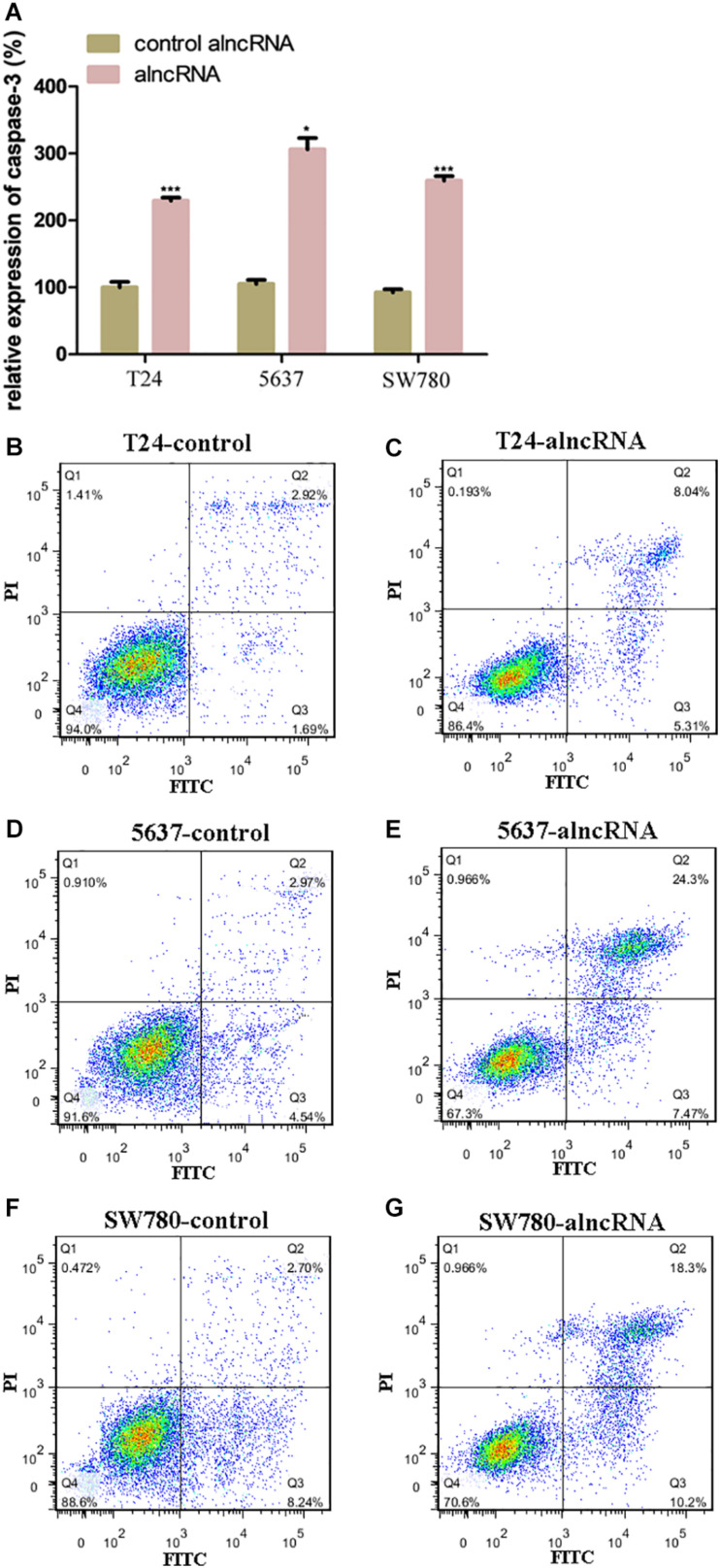
The alncRNA induced the cell apoptosis of BCa cell lines. **(A)** The relative expression of caspases-3 in BCa cell lines with or without alncRNA. **(B,C)** The alncRNA induced apoptosis in BCa cell line (T24) by flow cytometry analysis following Annexin V and 7-AAD staining. **(D,E)** The alncRNA induced apoptosis in BCa cell line (5637) by flow cytometry analysis following Annexin V and 7-AAD staining. **(F,G)** The alncRNA induced apoptosis in BCa cell line (SW780) by flow cytometry analysis following Annexin V and 7-AAD staining. *p < 0.05 and ***p < 0.001.

Prior to this, we have shown that the alncRNA can be used to interfere with the oncogenic signaling pathways. At present, we investigated the effect of the alncRNA on inducing cell apoptosis. In our work, we used caspase-3 ELISA assay and flow cytometry assay to evaluate the level of apoptosis induced by the alncRNA. First, apoptosis levels of BCa cell lines (T24, 5637, and SW780) were measured with caspase-3 ELISA assay (Figure 6A) and flow cytometry assay (Figures 6B–G). Next, after the stable expression of alncRNA in the BCa cells (T24, 5637 and SW780), we evaluated the apoptosis levels of these cells with caspase-3 ELISA assay (Figure 6A) and flow cytometry assay (Figures 6B–G). According to these experimental results, we concluded that the alncRNA we constructed could effectively induce cell apoptosis of BCa cells. Previous study has reported that β-catenin inhibited apoptosis by inhibiting the action of cleaved-PARP –apoptotic protein and caspase-3 protein (Jeon et al., 2011; Yang et al., 2015). Therefore, the alncRNA we constructed can promote apoptosis of the BCa cells by influencing the relevant pathways of β-catenin.

### The alncRNA We Constructed Inhibited Cell Proliferation in BCa Cells

**FIGURE 7:**
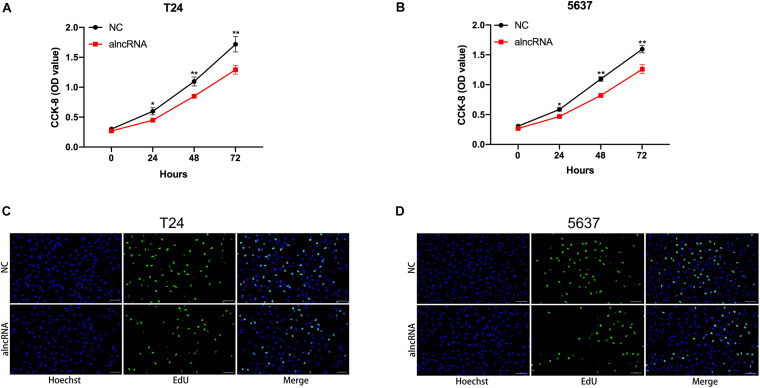
The alncRNA inhibited cell proliferation in BCa cells **(A)** The growth curves of T24 cells infected with or without alncRNA were determined using CCK-8 assay. **(B)** The growth curves of 5637 cells infected with or without alncRNA were determined using CCK-8 assay. **(C)** The proliferation of T24 cells infected with or without alncRNA were also determined using the EDU incorporation assay. **(D)** The proliferation of 5637 cells infected with or without alncRNA were also determined using the EDU incorporation assay. *p < 0.05 and **p < 0.01.

Next, we investigated whether the alncRNA can effectively inhibit cell proliferation in BCa cell lines (T24 and 5637). C-myc and cyclin D1 are well-known cell cycle regulator factors, which may serve as oncogenic factors and promote the cell proliferation of BCa cells. In our study, we planned to use this alncRNA to inhibit the signaling pathways associated with C-myc and cyclin D1 and tried to suppress tumor proliferation in this way. In the previous experimental results, we have demonstrated that the alncRNA we constructed indeed down-regulate the expression of C-myc and cyclin D1. Now, we planned to verify that this approach does inhibit cell proliferation in BCa cells (T24, 5637, and SW780). CCK-8 assay (Figures 7A,B) and edu assay (Figures 7C,D) were used to determine the cell proliferation levels of BCa cell lines T24 and 5637 in our study. First, we determined the levels of cell proliferation in BCa cell lines T24 and 5637. And then, after the stable expression of the alncRNA in the BCa cell lines, the cell proliferation of these cell lines will be measured again by using CCK-8 assay and edu assay. By comparison, we concluded that this alncRNA we constructed can effectively inhibit cell proliferation.

### The alncRNA We Constructed Inhibited Cell Migration in BCa Cells

**FIGURE 8:**
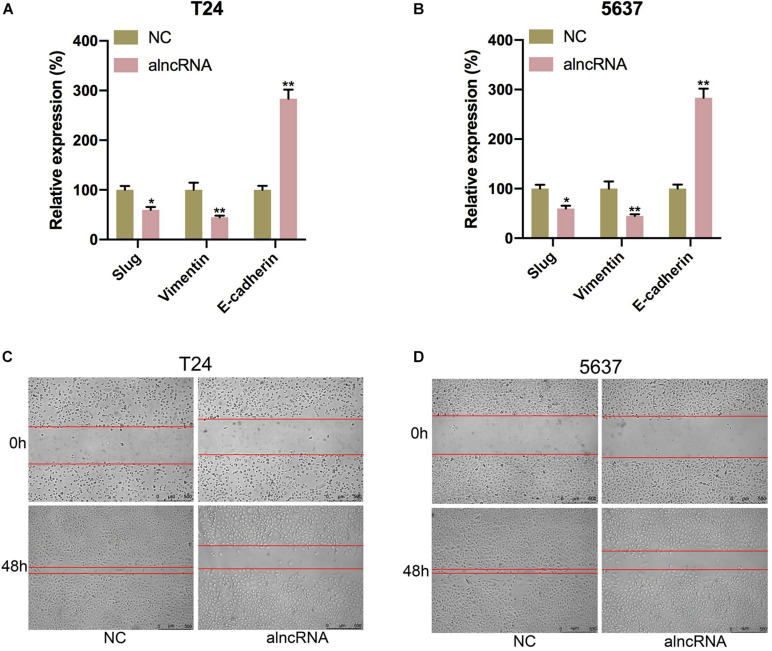
The alncRNA we constructed inhibited cell migration in BCa cells. **(A)** The relative expression of E-cadherin, vimentin and slug in T24 cell with or without alncRNA. **(B)** The relative expression of E-cadherin, vimentin and slug in 5637 cell with or without alncRNA. **(C)** The relative rate of cell migration was calculated in T24 cell infected with or without alncRNAs using the wound-healing assay. **(D)** The relative rate of cell migration was calculated in 5637 cell infected with or without alncRNAs using the wound-healing assay. *p < 0.05 and **p < 0.01.

Last, we tried to elucidate whether the alncRNA acted as a cell migration suppressor in BCa cells. Within the BCa cells, the targeted miRNAs miRNA-495 and miRNA-940, as well as β-catenin, were reported to induce cancer cell migration and invasion by inducing epithelial-mesenchymal transition (EMT) (Sun et al., 2016; Chen et al., 2017; Zhong et al., 2017; Wang et al., 2018). We supposed that this alncRNA can inhibit the cell migration of the BCa cells by inhibiting EMT. To validate our hypothesis, we investigated the relationship between the expression levels of mesenchymal markers (vimentin, slug) and epithelial marker (E-cadherin) and the cell migration levels of BCa cell (T24, 5637, and SW780) under the function of the alncRNA. qRT-PCR was used to measure the expression levels of E-cadherin, vimentin and slug, while wound healing assay was used to evaluate the cell migration activity in our study. We found that when the alncRNA was present, the expression of vimentin and slug were decreased obviously, while the expression of E-cadherin was increase obviously (Figures 8A,B). In addition, we also found that the cell migration of BCa cells was significantly reduced when the alncRNA was present (Figures 8C,D).

## Discussion

Nucleic acid aptamer is a small series of nucleotide sequences or short polypeptides to be screened *in vitro*, and it can bind to the corresponding ligands with high affinity and strong specificity. In our study, we concatenate the apatamers of β-catenin and NF-κB together to form the module1 of the alncRNA. The relationship between the aptamer and the protein ligand was taken advantage of by us to make the alncRNA we constructed specifically bindβ-catenin and NF-κB, respectively, and inhibit their oncogenic role within the BCa cells.

As one of the important regulatory factors, micoRNAs can reverse the expression of the target genes by inhibiting the translation of the target genes or degrading the mRNAs of the target genes. However, in the course of the actual regulatory process, some non-coding RNAs also have the binding site of miRNAs, which act as the miRNA sponge within the cells. These non-coding RNA can prevent miRNAs from inhibiting the target genes, thus increasing the expression level of the target genes. According to this principle, we designed module 2 of alncRNA.

In our study, we effectively combined the sequences of the oncogenic transcription factors aptamers and the sequences of the oncogenic miRNAs to construct an anti-cancer alncRNA molecule. The alncRNA we constructed inhibited the malignant phenotype of the BCa cells by binding to the oncogenic factors specifically. Our study also demonstrated that the malignant phenotype of the BCa cells can indeed be effectively inhibited by constructing the form of alncRNA molecules. At the same time, the inhibitory effect of alncRNA was better than CRISPR dCas9-KRAB. This strategy of inhibiting the progression of BCa may also provide novel ideas for the treatment of other malignant tumors.

However, there are still some deficiencies in our works. Firstly, our work primarily focused on the level of eukaryotic cells rather than demonstrating the effectiveness of the alncRNA *in vivo*. After all, we have not been able to demonstrate the validity of the alncRNA *in vivo*, which is not convincing enough for the suitability of this strategy for the clinical treatment. Secondly, we mainly use transfection to get the alncRNA into the cancer cells in our work. This approach, however, cannot make the alncRNA stable in cells over the long term. Therefore, the use of other more effective approach that enable the alncRNA to be stably retained in the cells will be our focus in the future.

In summary, we successfully constructed the alncRNA which can down-regulate the oncogenic factors in the BCa cells.

## Data Availability Statement

The raw data supporting the conclusions of this article will be made available by the authors, without undue reservation, to any qualified researcher.

## Author Contributions

LY, QuZ, and AL designed the project and wrote the manuscript. LY, QuZ, AL, BM, ZZ, JL, LL, and SZ performed experiments and data analysis. QiZ and YG supervised the project and provided financial support for the project. All authors contributed to the article and approved the submitted version.

## Conflict of Interest

The authors declare that the research was conducted in the absence of any commercial or financial relationships that could be construed as a potential conflict of interest.

## References

[B1] Bracken C. P. Scott H. S. Goodall G. J. (2016). A network-biology perspective of microRNA function and dysfunction in cancer. *Nat. Rev. Genet.* 17 719–732. 10.1038/nrg.2016.134 27795564

[B2] Chen H. Wang X. Bai J. He A. (2017). Expression, regulation and function of miR-495 in healthy and tumor tissues. *Oncol. Lett.* 13 2021–2026. 10.3892/ol.2017.5727 28454357 PMC5403365

[B3] Culler S. J. Hoff K. G. Smolke C. D. (2010). Reprogramming cellular behavior with RNA controllers responsive to endogenous proteins. *Science* 330 1251–1255. 10.1126/science.1192128 21109673 PMC3171693

[B4] Djebali S. Davis C. A. Merkel A. Dobin A. Lassmann T. Mortazavi A. (2012). of transcription in human cells. *Nature* 489 101–108. 10.1038/nature11233 22955620 PMC3684276

[B5] Haberle V. Stark A. (2018). Eukaryotic core promoters and the functional basis of transcription initiation. *Nat. Rev. Mol. Cell Biol.* 19 621–637. 10.1038/s41580-018-0028-8 29946135 PMC6205604

[B6] Hanahan D. Weinberg R. A. (2011). Hallmarks of cancer: the next generation. *Cell* 144 646–674. 10.1016/j.cell.2011.02.013 21376230

[B7] Jeon H. G. Yoon C. Y. Yu J. H. Park M. J. Lee J. E. Jeong S. J. (2011). Induction of caspase mediated apoptosis and down-regulation of nuclear factor-kappaB and Akt signaling are involved in the synergistic antitumor effect of gemcitabine and the histone deacetylase inhibitor trichostatin A in human bladder cancer cells. *J. Urol.* 186 2084–2093. 10.1016/j.juro.2011.06.053 21944112

[B8] Kamat A. M. Hahn N. M. Efstathiou J. A. Lerner S. P. Malmstrom P. U. Choi W. (2016). Bladder cancer. *Lancet* 388 2796–2810. 10.1016/s0140-6736(16)30512-30518 27345655

[B9] Kluth L. A. Black P. C. Bochner B. H. Catto J. Lerner S. P. Stenzl A. (2015). Prognostic and prediction tools in bladder cancer: a comprehensive review of the literature. *Eur. Urol.* 68 238–253. 10.1016/j.eururo.2015.01.032 25709027

[B10] Kwak H. Hwang I. Kim J. H. Kim M. Y. Yang J. S. Jeong S. (2009). Modulation of transcription by the peroxisome proliferator-activated receptor delta–binding RNA aptamer in colon cancer cells. *Mol. Cancer Ther.* 8 2664–2673. 10.1158/1535-7163.mct-09-0214 19723884

[B11] Lee H. K. Choi Y. S. Park Y. A. Jeong S. (2006). Modulation of oncogenic transcription and alternative splicing by beta-catenin and an RNA aptamer in colon cancer cells. *Cancer Res.* 66 10560–10566. 10.1158/0008-5472.can-06-2526 17079480

[B12] Liu Y. Huang W. Zhou D. Han Y. Duan Y. Zhang X. (2013). Synthesizing oncogenic signal-processing systems that function as both “signal counters” and “signal blockers” in cancer cells. *Mol. Biosyst.* 9 1909–1918. 10.1039/c3mb70093c 23619462

[B13] Liu Y. Zhan Y. Chen Z. He A. Li J. Wu H. (2016). Directing cellular information flow via CRISPR signal conductors. *Nat. Methods* 13 938–944. 10.1038/nmeth.3994 27595406

[B14] Marta G. N. Hanna S. A. Gadia R. Correa S. F. Silva J. L. Carvalho Hde A. (2012). The role of radiotherapy in urinary bladder cancer: current status. *Int. Braz. J. Urol.* 38 144–153. 10.1590/s1677-55382012000200002 22555038

[B15] Racioppi M. D’Agostino D. Totaro A. Pinto F. Sacco E. D’Addessi A. (2012). Value of current chemotherapy and surgery in advanced and metastatic bladder cancer. *Urol. Int.* 88 249–258. 10.1159/000335556 22354060

[B16] Rupaimoole R. Slack F. J. (2017). MicroRNA therapeutics: towards a new era for the management of cancer and other diseases. *Nat. Rev. Drug Discov.* 16 203–222. 10.1038/nrd.2016.246 28209991

[B17] Siegel R. L. Miller K. D. Jemal A. (2017). Cancer statistics, 2017. *CA Cancer J. Clin.* 67 7–30. 10.3322/caac.21387 28055103

[B18] Sofra M. Fei P. C. Fabrizi L. Marcelli M. E. Claroni C. Gallucci M. (2013). Immunomodulatory effects of total intravenous and balanced inhalation anesthesia in patients with bladder cancer undergoing elective radical cystectomy: preliminary results. *J. Exp. Clin. Cancer Res.* 32:6. 10.1186/1756-9966-32-36 23374147 PMC3577511

[B19] Sun Y. Guan Z. Liang L. Cheng Y. Zhou J. Li J. (2016). NF-kappaB signaling plays irreplaceable roles in cisplatin-induced bladder cancer chemoresistance and tumor progression. *Int. J. Oncol.* 48 225–234. 10.3892/ijo.2015.3256 26647959

[B20] Tan M. Mu X. Liu Z. Tao L. Wang J. Ge J. (2017). microRNA-495 promotes bladder cancer cell growth and invasion by targeting phosphatase and tensin homolog. *Biochem. Biophys. Res. Commun.* 483 867–873. 10.1016/j.bbrc.2017.01.019 28069380

[B21] Tetsu O. McCormick F. (1999). Beta-catenin regulates expression of cyclin D1 in colon carcinoma cells. *Nature* 398 422–426. 10.1038/18884 10201372

[B22] Wang R. Wu Y. Huang W. Chen W. (2018). MicroRNA-940 targets INPP4A or GSK3beta and activates the Wnt/beta-Catenin pathway to regulate the malignant behavior of bladder cancer cells. *Oncol. Res.* 26 145–155. 10.3727/096504017X14902261600566 28337959 PMC7844674

[B23] Wong Y. N. S. Joshi K. Pule M. Peggs K. S. Swanton C. Quezada S. A. (2017). Evolving adoptive cellular therapies in urological malignancies. *Lancet Oncol.* 18 e341–e353. 10.1016/s1470-2045(17)30327-30323 28593860

[B24] Yang T. Shi R. Chang L. Tang K. Chen K. Yu G. (2015). Huachansu suppresses human bladder cancer cell growth through the Fas/Fasl and TNF- alpha/TNFR1 pathway in vitro and in vivo. *J. Exp. Clin. Cancer Res.* 34:21 10.1186/s13046-015-0134-139 25887782 PMC4354737

[B25] Zhan H. Xie H. Zhou Q. Liu Y. Huang W. (2018). Synthesizing a genetic sensor based on CRISPR-Cas9 for specifically killing of P53-deficient cancer cells. *ACS Synth. Biol.* 7 1798–1807. 10.1021/acssynbio.8b00202 29957992

[B26] Zhong W. Chen S. Qin Y. Zhang H. Wang H. Meng J. (2017). Doxycycline inhibits breast cancer EMT and metastasis through PAR-1/NF-kappaB/miR-17/E-cadherin pathway. *Oncotarget* 8 104855–104866. 10.18632/oncotarget.20418 29285218 PMC5739605

